# Retrospective Data Analysis and Literature Review for a Development of Enhanced Recovery after Surgery Pathway for Anterior Cervical Discectomy and Fusion

**DOI:** 10.7759/cureus.6930

**Published:** 2020-02-10

**Authors:** Fassil B Mesfin, Stanley Hoang, Michael Ortiz Torres, Ruben Ngnitewe Massa'a, Raul Castillo

**Affiliations:** 1 Neurosurgery, University of Missouri, Columbia, USA; 2 Neurosurgery, University of Missouri School of Medicine, Columbia, USA; 3 Anesthesiology, University of Missouri Health Care and University of Missouri School of Medicine, Columbia, USA

**Keywords:** enhanced recovery after surgery, anterior cervical discectomy and fusion, multimodal analgesia

## Abstract

Objective

While enhanced recovery after surgery (ERAS) protocols are associated with shorter length of stay and improved outcomes in multiple surgical specialties, its application to spine surgery has been limited. Anterior cervical discectomy and fusion (ACDF) is a common spinal procedure with a relative efficacy and safety profile that makes it suitable for the application of ERAS principles. Reviewing our outcomes and practice and incorporating evidence-based clinical studies, we propose the development of an ERAS pathway for ACDF.

Methods

This is a retrospective review of ACDF cases performed at a single institution by a single surgeon from 2014 to 2017. Primary outcome measures included length of stay, complications, and 30-day readmission rates. The 1- and 2-level and the 3- and 4-level groups were also each consolidated into a single cohort for comparison. A comprehensive review of evidence-based literature pertaining to ACDF was then performed. Best-practice recommendations derived from the literature were incorporated into the proposed ERAS protocol.

Results

In this series of 75 1-level, 77 2-level, 44 3-level and 20 4-level ACDF procedures, the average surgical time (minutes) was 68, 90, 118 and 141; length of stay (days) was 1, 1, 1.4, and 1.7; drain usage (%) was 1.3, 2.6, 13.6 and 10; and 30-day readmission rates (%) were 2.7, 3.9, 4.5, and 15, respectively. Combining the 1- and 2-level as a single group and 3- and 4-level as another cohort, the 3- and 4-level cohort had a significantly higher rate of drain usage and estimated blood loss (EBL) but there was not a difference in length-of-stay, complications or 30-day readmission rates.

Conclusions

Given the relative equivalent safety profile between 1- and 2-level as compared to 3- and 4-level ACDF, the proposed ERAS pathway can be applied to all patients, and not just restricted to 1-level or 2-level ACDF. Taking into account feasibility parameters as deduced from a review of institutional outcomes, this pathway can streamline same-day discharge and improve the patient experience. Its success will be predicated on an iterative improvement process deriving from optimal prospective outcome measurements.

## Introduction

Enhanced recovery after surgery (ERAS) is a multimodal, evidence-based approach that aims to modulate the surgical stress response to accelerate post-operative recovery and reduce morbidity [1]. Anterior cervical discectomy and fusion (ACDF), with its relative efficacy and low morbidity, is increasingly being performed as an outpatient procedure [2]. However, partly due to concern about the higher complication rates associated with multi-level ACDF, in a recent meta-analysis of outpatient ACDF procedures, it was found that almost two-thirds of the outpatient procedures were a single-level, with virtually none undergoing 3- or 4-level ACDF [3,4]. Consequently, we are interested in describing whether there is a difference in our complication rates between 1- and 2-level compared to 3- and 4-level ACDF as to determine whether it is advisable to include 3- and 4-level ACDF in an ERAS protocol. Reviewing our data and outcomes, while incorporating a comprehensive number of evidence-based clinical studies, we developed an ERAS pathway for ACDF.

## Materials and methods

Retrospective data review

Following institutional review board approval, a retrospective review of clinical data was performed on patients that had undergone 1-, 2-, 3- or 4-level ACDF by a single attending physician from 2014 to 2017 for treatment of degenerative cervical spine disease. Exclusion criteria were: less than 30-day follow-up, corpectomy, and those with indications resulting from trauma, infection, or neoplasm. Primary outcome measurements included length of stay, complications, and 30-day readmission rates. Statistical analysis was performed via GraphPad Prism 7 using unpaired 2-tailed t-tests with unequal variances. A p-value < 0.05 was defined as statistically significant.

ERAS pathway design

In addition to utilizing the data conditions from the retrospective data review, a literature review of established ERAS pathways was conducted using PubMed for English articles published before October 2018. The initial focus was broad, and subsequently narrowed to spinal procedures and ACDF. Since there has not been a published ERAS pathway for ACDF, articles pertaining to trial designs, techniques or recommendations with evidence suggesting improvement in post-operative course were selected. Based on available evidence, recommendations were compiled and, together with our outcome data, an ERAS protocol for ACDF is proposed.

## Results

1-level ACDF

For patients who underwent 1-level ACDF, there were 75 patients, 49% were males, with a mean age of 51.8 and a mean BMI of 31.5 (Table 1). On average, surgical time was 68 minutes, with an estimated blood loss (EBL) of 17 ml and hospital length of stay (LOS) of one day. Only one patient (1.3%) had placement of a surgical drain, which was removed on postoperative day (POD) 1. Most patients had an unremarkable post-operative course requiring 1-2 days of admission but three patients (4%) had eventful hospital stays, two of which were directly related to the procedure. One patient had dysphagia immediately post-op that improved with steroid treatment. The second patient developed new onset of upper extremity numbness and weakness that self-resolved after 24 hours. The third patient had urinary retention related to traumatic urinary catheter placement. All three patients were discharged home on POD 2 and seen in follow-up with expected recovery. One patient (1.3%) developed a superficial wound infection that was successfully treated with a 14-day course of oral antibiotics. Two patients (2.7%) were readmitted within 30 days following discharge - one was due to chronic obstructive pulmonary disease (COPD) exacerbation and the other was due to gastroenteritis, both of which were managed medically.

2-level ACDF

For patients who underwent 2-level ACDF, there were 77 patients, 43% were males, with a mean age of 55 and a mean BMI of 29.1 (Table 1). On average, surgical time was 90 minutes, with an EBL of 24.8 ml and LOS of 1 day. Two patients (2.6%) had placement of a drain, both of which were removed on POD 1. Two patients (2.6%) had eventful hospital stays. One was a COPD exacerbation and the other a compressive subplatysmal hematoma, which required takeback to the operating room for evacuation. Both patients were discharged within 72 hours post-operatively. There were no wound infections. Three patients (3.9%) were readmitted within 30 days following discharge for medical reasons: hyponatremia, suicidal ideation and acute bronchitis exacerbation.

3-level ACDF

For patients who underwent 3-level ACDF, there were 44 patients, 50% were males, with a mean age of 57 and a mean BMI of 31 (Table 1). On average, surgical time was 118 minutes, with an EBL of 33 ml and LOS of 1.4 days. Six patients (13.6%) had placement of surgical drain, all of which were removed on POD 1. All patients had uneventful hospital stays. One patient (2.3%) had a superficial wound infection that was treated with a 10-day course of oral antibiotics. Two patients (4.5%) were readmitted within 30 days following discharge for dysphagia, which improved with steroids.

4-level ACDF

For patients who underwent 4-level ACDF, there were 20 patients, 55% were males, with a mean age of 59 and a mean BMI of 30 (Table 1). On average, surgical time was 141 minutes, with an EBL of 47 ml and LOS of 1.7 days. Two patients (10%) had drain placement; one was removed on POD 1 and the other on POD 2. Four patients (20%) had eventful hospital stays consisting of significant dysphagia requiring steroids. Three patients (15%) were readmitted within 30 days following discharge. One patient had a wound infection associated with dehiscence that required returning to the operating room for wound revision, followed by a six-week course of intravenous antibiotics. The second patient had a subcutaneous hematoma and dysphagia that was managed with steroids. The last patient had altered mental status and delirium that were managed medically.

**Table 1:** Patient demographics and outcomes for each ACDF level. Values are presented as the number of patients and percentages. Mean values are reported as the mean ± standard deviation. ACDF: Anterior cervical discectomy and fusion; BMI: Body mass index; EBL: Estimated blood loss; LOS: Hospital length of stay.

Parameter	1-level		2-level	3-level	4-level
No. of patients	75		77	44	20
Mean age (years)	51.8 ± 12.1		55 ± 10.2	57.2 ± 8.8	59.8 ± 8.1
Male sex (%)	37 (49%)		43 (56%)	22 (50%)	11 (55%)
BMI	31.5 ± 8		29.1 ± 5.8	31.3 ± 6.5	30.3 ± 7.8
Drain	1 (1.3%)		2 (2.6%)	6 (13.6%)	2 (10%)
Surgery time (mins)	68 ± 14.7		90 ± 17.5	118 ± 21	141 ± 21
EBL (ml)	17.4 ± 8.3		24.8 ± 17.7	33 ± 20	47 ± 33
LOS (days)	1.4 ± 0.7		1.4 ± 0.7	1.5 ± 1	1.5 ± 0.7
Eventful hospital course	3 (4%)		2 (2.6%)	0	4 (20%)
Wound infection	1 (1.3%)		0	1 (2.3%)	1 (5%)
30-day re-admission	2 (2.7%)		3 (3.9%)	2 (4.5%)	3 (15%)

1- and 2-level vs. 3- and 4-level

Data for 1- and 2-level were pooled into a single cohort while data for 3- and 4-level were pooled into a second cohort for comparison (Table 2). Demographic variables including gender and BMI were equivalent, except that the 3- and 4-level group was older. In addition, the 3- and 4-level group had a significantly higher rate of drain usage and EBL but there was not a difference in length-of-stay, eventful hospital course, wound infection or 30-day re-admission rates.

**Table 2:** Comparison between 1- and 2-level vs. 3- and 4-level ACDF. Values are presented as the number of patients and percentages. Mean values are reported as the mean ± standard deviation. p-values are based off a 0.05 level of significance. * Indicates a statistically significant value. ACDF: Anterior cervical discectomy and fusion; BMI: Body mass index; EBL: Estimated blood loss; LOS: Hospital length of stay.

Parameter	1- and 2-level	3- and 4-level	p-value
No. of patients	152	64	
Mean age (years)	53.7 ± 11.3	57.3 ± 8.8	0.02^*^
Male sex (%)	80 (53%)	33 (52%)	0.8
BMI	30.3 ± 7.1	31 ± 6.9	0.51
Drain	3 (1.9%)	8 (12.3%)	0.001^*^
Surgery time (mins)	76.7 ± 19.6	125.3 ± 23.7	0.0001^*^
EBL (ml)	21.1 ± 14.3	37.5 ± 25.5	0.55
LOS (days)	1.4 ± 0.7	1.5 ± .9	1
Eventful hospital course	5 (3.3%)	4 (6.3%)	0.3
Wound infection	1 (0.7%)	2 (3.1%)	0.14
30-day re-admission	5 (3.3%)	5 (7.8%)	0.14

## Discussion

A retrospective review of our clinical data over the last four years demonstrates a pattern of practice that is amenable to implementation of an enhanced recovery protocol. For all levels of ACDF, most of our patients are currently being kept overnight. Nevertheless, we make minimal use of drains and our surgical time for 1- and 2-level procedures is less than two hours while that of our 3- and 4-level procedures is slightly more than two hours. There was also not a significant difference in complication rates between 1- and 2- vs. 3- and 4-level procedures. Considering these findings, we believe that the proposed ERAS pathway can be applied to both single and multi-level ACDF, with the goals of accomplishing same-day discharge and improving patients’ outcomes.

Pre-operative

**Figure 1:**
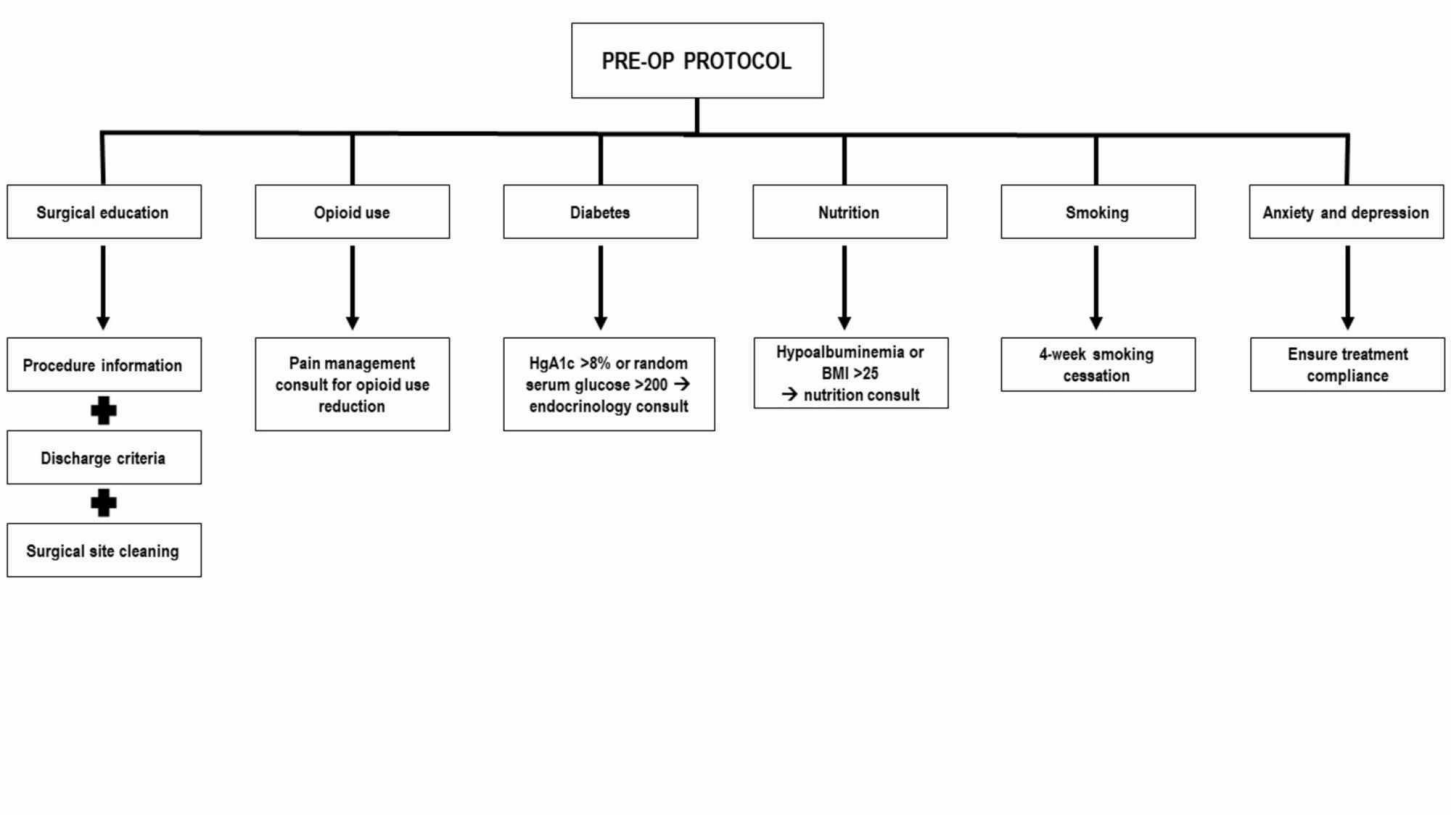
Pre-operative ERAS elements include surgical education, opioid use reduction, glycemic control, nutritional optimization, smoking cessation, and treatment of depression and anxiety. ERAS: Enhanced recovery after surgery

The pre-operative component of the ERAS protocol aims to optimize the patient’s physical and functional status and educate about the procedure and recovery (Figure 1) [5].

Surgical education

Patient education will include pre-admission testing, medication use, peri-operative eating and drinking, expected post-operative complications and discharge criteria [6]. For patients who have recently undergone epidural steroid injections, surgery will be scheduled at least three months from the date of injection to minimize infection risks. A retrospective database was used to compare postoperative infection rates within 90 days in patients who received a cervical epidural steroid injection (ESI) before undergoing ACDF stratified into three cohorts: ACDF within 3, between 3-6 months, and between 6-12 months following ESI [7]. Compared to controlled cohorts, patients who had an ESI within three months before ACDF had a significantly higher rate of postoperative infection. In addition, patients will be provided with surgical site cleansing education. A chlorhexidine gluconate (CHG) solution will be provided for washing the incision site for three consecutive nights before surgery [5]. The rate of compliance will be monitored by asking the patients about CHG use on day of surgery.

Opioid use

Chronic preoperative opioid use is associated with worse outcomes following ACDF [8]. In a prospectively collected questionnaire of 91 patients who underwent ACDF, the use of narcotics on a daily basis for more than six months before surgery was associated with worse outcomes and continued post-operative narcotics use [9]. In another study of 281 patients who underwent single-level ACDF, patients with pre-operative opioid use were less likely to return to work after one year [10]. Moreover, patients with more neck than arm pain were also less likely to improve in overall disability following surgery. Consequently, patients who use greater than 30 morphine equivalent dose (MED) for more than four weeks will be referred to pain management to address preoperative opioid reduction [5]. As simple opioid reduction or abstinence is not likely to happen without provision of viable alternatives to pain management, a multidisciplinary preoperative optimization program for chronic pain patients is necessary to achieve optimal reduction.

Diabetes

In patients undergoing spine surgery, diabetes, both insulin dependent (IDDM) and non-insulin dependent (NIDDM), is associated with higher infection rates, more complications, and greater medical costs [11]. In a retrospective analysis of 3726 ACDF cases, patients with NIDDM had higher rates of urinary tract infection and return to the operating room, while patients with IDDM had higher rates of reoperations, readmissions and length of stay. IDDM was also an independent predictor of increased 30-day readmissions [12]. Moreover, another study of 29 diabetic patients who underwent a single-level ACDF found them to have lower fusion rates, although there was not a significant difference in functional outcomes or quality of life [13]. As pre-operative Hemoglobin A1C (HbA1c) is a reliable measure of chronic diabetes control, patients with HbA1c > 8% or with random serum glucose > 200 mg/dL should have an endocrinology consult to achieve pre-operative glycemic control [5].

Nutrition

Nutritional optimization is an essential component of preoperative preparation. In a retrospective review of 3671 ACDF cases, of which 37% had preoperative albumin measurements, hypoalbuminemia was associated with major pulmonary and cardiac complications, reoperation and longer hospital stay [14]. On the other hand, obesity has not been found to be associated with less improvements in patient-reported outcomes and even morbidly obese patients showed significant improvements in pain, disability and quality of life [15]. A more recent analysis of 277 patients who underwent 1- and 2-level ACDF also showed that higher BMI patients had comparable outcomes, narcotic use, and hospital costs to those with lower BMI [4]. Therefore, although ACDF procedures should be considered for patients across the BMI spectrum, preoperative nutritional consultation and optimization is important to promote recovery, especially in patients with hypoalbuminemia and high BMI (>25) [7].

Smoking

Smoking cessation is generally advisable to optimize wound healing and improve outcomes. Nevertheless, in a retrospective review of 573 patients (156 smokers and 417 nonsmokers) with a minimum follow-up period of 24 months who underwent a single level ACDF with allograft and a locked cervical plate, no statistically significant difference in fusion rate was found [16]. Moreover, in a large retrospective review of ACDF patients who were either current smokers or non-current smokers, current smoking status or number of pack-years was not associated with increased risk of complication. However, people who have ever-smoked did have a higher risk of developing complications. Therefore, although the association of smoking and poor outcome in ACDF is not as evident as that found in other surgical procedures, a prior smoking history should be considered a risk factor in patients undergoing ACDF [17]. In active smokers, a mandatory smoking-cessation period of four weeks prior to surgery with appropriate aids and counseling should be considered [5].

Anxiety and depression

Patients with a greater degree of depression pre-operatively may have lower improvements in postoperative quality of life outcomes [18]. In those with depression, pretreatment with antidepressants before surgery can improve perception of pain and functional disability. In patients with anxiety, treatment before surgery results in significant reduction in postoperative neck pain [19]. On the other hand, a more recent study of 52 patients who underwent 1- or 2-level ACDF failed to demonstrate a correlation between preoperative SF-12 Mental Component Summary (MCS) scores and improvement in Neck Disability Index (NDI), SF-12 Physical Component Summary (PCS), or neck and arm pain [20]. While more research is necessary, for patients with an established psychiatric diagnosis, treatment compliance will be ensured prior to surgery to optimize recovery.

Peri-operative

**Figure 2:**
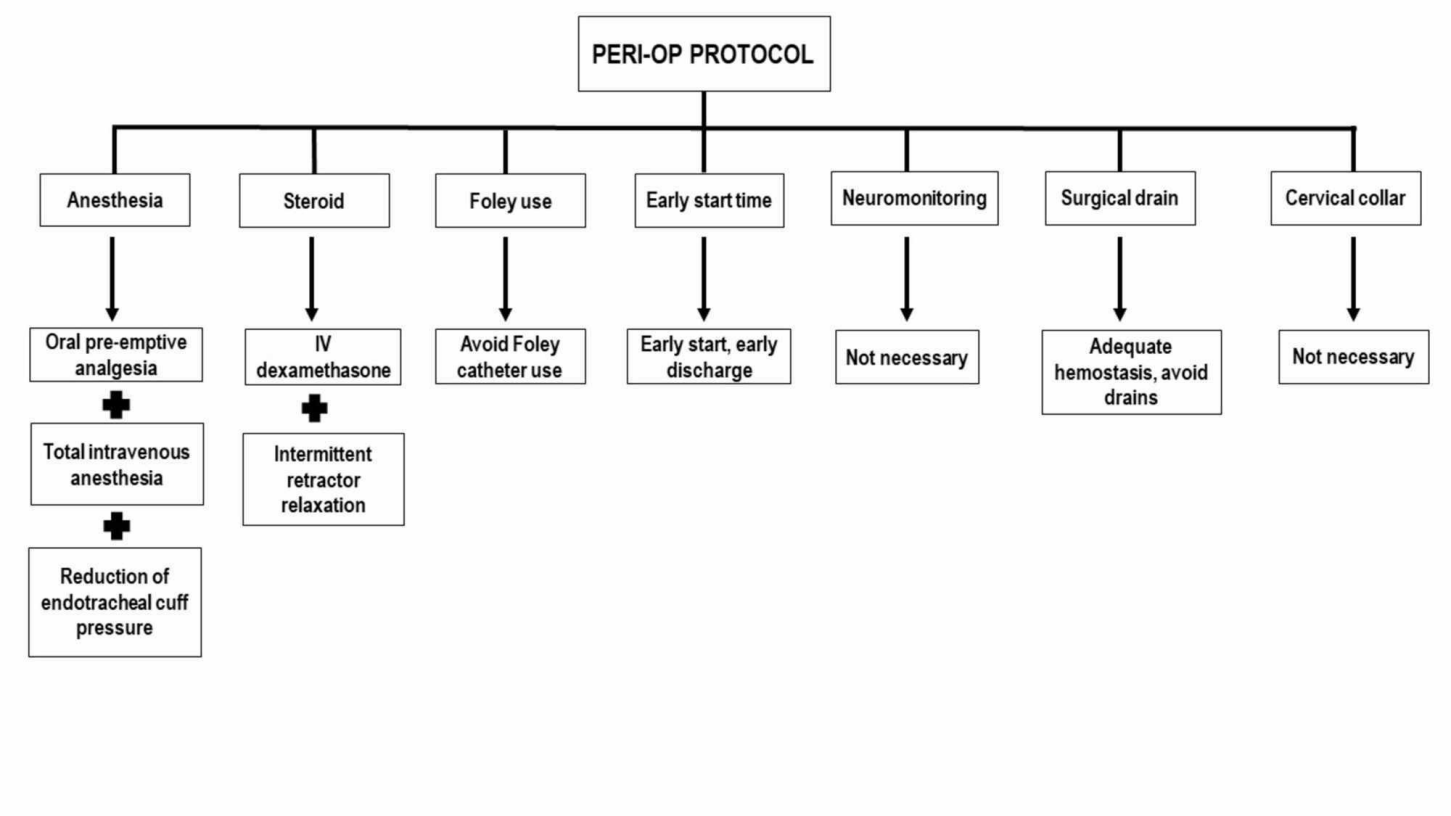
Peri-operative ERAS elements include oral pre-emptive analgesia and total intravenous anesthesia, IV dexamethasone to reduce swelling, Foley avoidance, and meticulous hemostasis to avoid surgical drains. ERAS: Enhanced recovery after surgery

The peri-operative period begins from the day of surgery until transfer to the Post-Anesthesia Care Unit (PACU). The goal here is to optimize anesthetic and surgical interventions to ensure optimal convalescence (Figure 2).

Anesthesia

Oral pre-emptive analgesia to improve post-operative nausea will be provided in the pre-op holding area. This will include acetaminophen (1 g), gabapentin (100 mg), tramadol (100 mg) and scopolamine patch (1.5 mg). Total intravenous anesthesia technique will be used preferentially while minimizing intravenous opioid [21]. Antibiotic prophylaxis will be administered. To reduce recurrent laryngeal nerve palsy, the anesthesiologists, following placement of surgical retractors, will deflate the endotracheal cuff and then re-inflate until a seal is obtained and when possible, reduce the endotracheal cuff pressure to below 20 mm Hg [22].

Steroid

Intravenous dexamethasone (0.2 mg/kg) will be given prior to surgical incision to reduce post-operative dysphagia. While the evidence for steroid use is still limited by risk of bias and a small number of studies, systematic reviews are generally in favor of steroid use. A recent review found that the incidence and severity of dysphagia and pre-vertebral soft-tissue swelling are generally lower in the steroid group, while airway compromise and LOS were inconclusive [23]. Complications related to steroid use were rare and fusion rates were comparable.

Foley use

Patients will be encouraged to void before anesthesia induction and Foley catheter will not be placed in those undergoing 1- or 2-level ACDF as the surgical time is usually less than two hours. For 3- and 4-level ACDF, Foley placement is determined on a case-by-case basis, and will be removed immediately postop, if placed. Avoiding Foley use will encourage early mobilization and reduce the risk of urinary tract infection [24].

Early start time

All cases will be scheduled mornings and early afternoon start to facilitate same-day discharge. A retrospective review of 106 patients undergoing ACDF was stratified into two cohorts based on surgical start time: first of the day before 9 am (early cohort), and later in the day (late cohort) [25]. The late cohort was more likely to stay overnight (45% of the early cohort were discharged on the day of surgery, as compared to only 26% of the late cohort).

Neurological monitoring

Intraoperative neurological monitoring will not be used. In a large retrospective review of a national database of patients who underwent a single-level ACDF, neuromonitoring did not lead to a reduction in neurological complications but was associated with an increase in total payments for the procedure and for hospitalization [26].

Surgical drain

Drains will not routinely be placed, as meticulous attention is paid to hemostasis at the end of the procedure. In a study of 43 patients who underwent one-level ACDF divided into two groups with and without drain placement, CT obtained at POD 1 did not show evidence of postoperative hematoma in any of the patients [27]. The group without drains also reported a milder pain level. In a retrospective study of 321 patients undergoing multi-level ACDF, 58 (18%) patients had subfascial drains placed based on surgeon preference [27]. Drain use in this study cohort was not associated with a decreased incidence of post-operative hematoma or surgical site infections; on the contrary, drain usage was associated with a higher likelihood for a post-operative blood transfusion and an almost two-fold increase in LOS. At the same time, it is important to ensure hemostasis of major vessels such as superior and inferior thyroid arteries, external jugular vein and prevertebral venous plexus during surgery.

Cervical collar

While there is some contention that cervical collar can reduce post-operative pain or rates of non-fusion, we generally have not used cervical collar due to concern for skin breakdown, difficulty swallowing, long-term sensory compromise, and general immobility [28]. In a prospective randomized control trial investigating the use of cervical collars following 1- and 2-level ACDF for six weeks postoperatively, there was not a statistically significant advantage as measured by Neck Disability Index scores at one-year, one-year fusion rates, and six-month subsidence rates. As a result, cervical collar will not be recommended.

Post-operative

**Figure 3:**
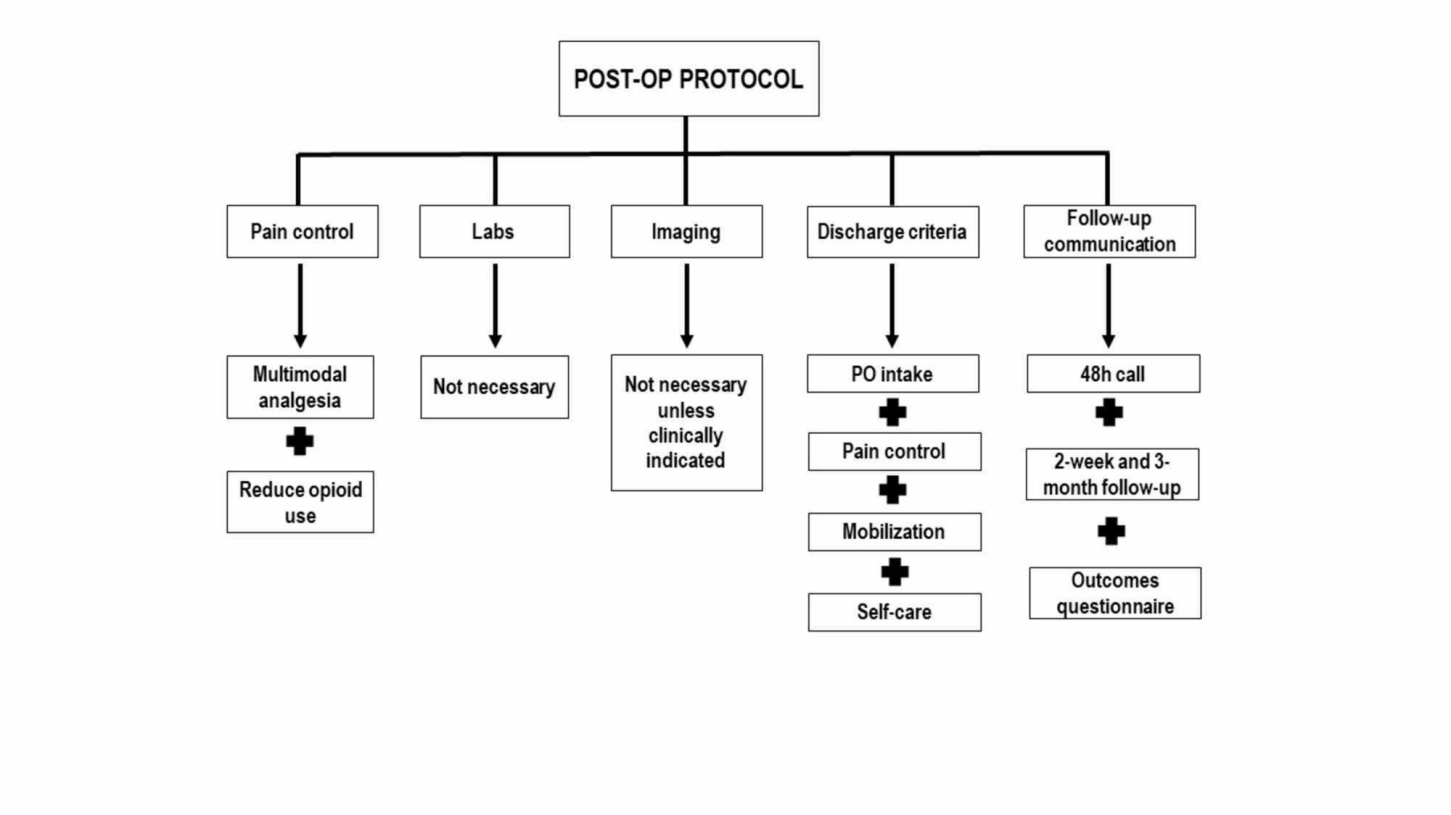
Post-operative ERAS elements include multimodal analgesia, minimal use of labs and imaging, meeting of discharge criteria and follow-up communication. ERAS: Enhanced recovery after surgery

The post-operative period includes care in the PACU and follow-up after discharge. Emphasis is also placed on direct patient communication after leaving the hospital to ensure continuity of care (Figure 3) [5].

Pain control

Post-operative pain control will employ preemptive and multimodal analgesia instead of patient-controlled analgesia and avoid the use of intravenous opioid. By inhibiting central autonomic hyperactivity with the use of nonsteroidal anti-inflammatory drugs, anti-convulsants, and acetaminophen, pre-emptive analgesia has shown improvement in immediate post-operative pain, anxiety and self-care. Post-operatively, we will use oral tramadol and acetaminophen until every discharge, unless a patient has known hepatic dysfunction [5]. Oral narcotics will be used on an as-needed basis, while intravenous narcotics will be minimized. Patient-controlled analgesia will not be used as multimodal analgesia has been found to be associated with lower narcotic consumption, shorter inpatient stay and reduction in post-operative nausea.

Laboratory studies

As ACDF is associated with minimal blood loss, unless there is significant intraoperative hemorrhage, or the patient has risk factors for postoperative anemia, routine laboratories will not be obtained. In a retrospective analysis of 332 patients who underwent ACDF, postoperative labs were compared with preoperative values [29]. While there was a significant reduction in the postoperative hemoglobin after 1- or 2-level ACDF, no patients required a blood transfusion or demonstrated hemodynamic imbalance. Therefore, routine postoperative labs are unlikely to alter management.

Imaging studies

Routine post-operative radiographs will not be taken prior to discharge, unless there is significant clinical concern. A study compared two ACDF groups, in which one group (n = 109) received routine post-operative XR imaging and the second group (n = 113) received radiographs only when clinically indicated. Routine post-operative XR did not change clinical management or mandate revision surgery. All cases that required surgery or further imaging were identified based on clinical deterioration. The group without imaging also had a shorter LOS [30]. Thus, refraining from obtaining routine post-operative radiographs can minimize radiation to sensitive neck structures and reduce medical costs.

Discharge criteria

Following surgery, the patient will be observed in the PACU for at least six hours until discharge criteria are met. An observation period of about six hours is generally deemed adequate to detect severe complications that preclude same-day discharge. Discharge criteria will include toleration of oral intake, adequate pain control (numeric rating scale <5), prompt mobilization within two hours of PACU arrival, and demonstration of adequate social support and sufficiency for self-care [5].

Follow-up communication

After discharge from the hospital, a member of the surgical team will call the patient within 48 hours to discuss pain management, diet, mobility, wound care, available triage resources and planned appointments [5]. The patient will follow up with both the primary care physician and surgeon within two weeks and then again at three months. Clinical monitoring and questionnaire regarding outcomes will be administered during these visits.

## Conclusions

The primary goal of the proposed ERAS pathway is to achieve same-day discharge while improving outcomes for patients undergoing ACDF. Besides being a patient-centered approach and modulating the stress of the surgical procedure, the ERAS pathway could improve process that allows for prompt modification to each step of the protocol based on outcome data. The success of this protocol will depend on establishing a core team of nurses, surgeons, anesthesiologists and ancillary staff, as well as having a receptive institutional culture and the availability of resources.
